# Public Awareness and Perception of Risks Associated With Neck Manipulations in Southern Saudi Arabia

**DOI:** 10.7759/cureus.75420

**Published:** 2024-12-09

**Authors:** Shorog Althubait, Alhanouf O Alasmari, Najwa S Jurays, Ahlam M Abu-Hashrah, Olaa M Omaish, Taif K Alasmari, Thikra K Alasmari, Yusra A Alqasimi

**Affiliations:** 1 Neurology and Interventional Neuroradiology, College of Medicine, King Khalid University, Abha, SAU; 2 Faculty of Medicine, King Khalid University, Abha, SAU

**Keywords:** complications, cross-sectional study, educational campaigns, neck manipulation, neck pain, public awareness, southern saudi arabia, stroke

## Abstract

Introduction

Neck manipulations, including massages and self-cracking, are common practices, yet public awareness of their potential risks is poorly understood. This study explores public knowledge and awareness of the risks associated with neck manipulations in the Southern region of Saudi Arabia.

Methods

The study employed a descriptive cross-sectional design from May 2024 to July 2024, distributing a questionnaire to the adult population in the Southern Region. A convenience sampling technique was used for data collection. The data were initially entered into an Excel spreadsheet for cleaning and subsequently analyzed using SPSS version 27.

Results

Among the respondents, 154 (39.8%) reported experiencing neck pain, and 196 (50.6%) admitted to engaging in neck manipulation, primarily for comfort (100, 51.0%) or pain relief (35, 17.9%). Despite these practices, 271 (70.0%) were unaware of the serious complications that could result. Most participants performed neck manipulations themselves (39.3%), often learning techniques from social media (75.3%). Key complications identified included neck fractures (44.8%) and paralysis (40.5%), yet awareness of risks like stroke was limited (19.8%). A statistically significant association was found between age and neck pain (p = 0.001). Individuals aged 30-39 reported the highest prevalence of neck pain. By contrast, no significant association was observed between gender and neck pain (p > 0.05).

Conclusion

There is a critical public knowledge gap regarding neck manipulation complications in the Southern region of Saudi Arabia. The results underscore the necessity for targeted public health interventions to improve awareness and encourage safer practices.

## Introduction

Neck pain is a prevalent issue accompanied by significant comorbidities, impairment, and societal costs [1]. Neck manipulation is a commonly utilized form of treatment. Approximately 12% of adults in the United States and Canada seek chiropractic care annually, with 80% of these visits resulting in neck manipulation [2]. In the Kingdom of Saudi Arabia, the prevalence of individuals receiving neck massage or crack is 46.0% [3]. Neck manipulations are a common therapeutic approach to managing musculoskeletal problems, including cervical pain, headaches, and back pain [4]. Specifically, adjustments or manipulations of the upper spine have been linked to severe adverse outcomes, including cerebrovascular incidents, paralysis, rib fractures, herniated cervical discs, and compression of the vertebral artery [5]. Subsequent research has estimated stroke rates ranging from one in 100,000 to one in 2,000,000 cervical manipulations. Approximately 2% of all ischemic strokes are caused by this factor, which is considered one of the causes of stroke [6]. According to a study conducted by the Canadian Stroke Consortium, it was discovered that 28% of strokes were associated with cervical artery dissection. The findings of this study indicate a potential connection between neck manipulations and stroke [7]. Fortunately, the incidence of serious complications is generally considered to be low. By Senstad et al.'s research [8], side effects are categorized into two groups, common and uncommon reactions, which are determined by their frequency of occurrence [8]. Common reactions include local discomfort, headache, fatigue, and radiating pain. On the other hand, uncommon reactions involve less frequently reported symptoms such as dizziness, nausea, and hot skin [8]. In a study conducted in Saudi Arabia, approximately 20% of the participants reported experiencing different symptoms. Among these symptoms, dizziness was the most commonly reported (10.0%), followed by fatigue (9.0%), headache (6.0%), and weakness (4.2%). It should be noted that only a small portion of those experiencing symptoms required hospital visits (16.6%) [3].

Notably, the existing research on public knowledge and awareness regarding the complications linked to neck manipulations in the Southern region of Saudi Arabia lacks comprehensive information and understanding among the general population regarding the potential risks and complications associated with such procedures.

This cross-sectional study aims to evaluate the gap in knowledge and awareness within the general populace of the Southern region of Saudi Arabia concerning the possible complications associated with neck manipulations. The study seeks to determine deficiencies in knowledge and understanding while investigating the factors that might impact the public. 

## Materials and methods

This cross-sectional questionnaire survey was conducted in southern Saudi Arabia from May 2024 to July 2024. The study aims to assess public knowledge and awareness regarding complications associated with neck manipulations among the Saudi Arabian population residing in the southern region.

Sample size and sampling technique

The study's participants were recruited through volunteer sampling. The online survey questionnaire targeted individuals aged 18 years and above, with no restrictions based on sex. The survey was distributed via social media platforms and email. Participants eligible for inclusion were those aged 18 years and above and residing in the southern region of Saudi Arabia. Individuals below 18 years old or those living outside this region were excluded from the study. The sample size was calculated using Epi Info software (CDC, Atlanta, GA, USA), version 7.2.2.6, with a 95% confidence level, a 5% margin of error, and an expected frequency of 50%. This resulted in an estimated sample size of 383 participants.

Data collection tools and procedures

The survey was conducted in the southern Saudi Arabia region from June to July 2024. Before completing the questionnaire, the participants were required to provide consent. The questionnaire (see Appendix) comprised questions on sociodemographic details such as age, gender, marital status, education level, and employment status and assessed awareness about the complications associated with neck maneuvers. The questionnaire, validated by previous studies [3], consisted of 30 items spanning demographic details and specific questions on public knowledge and awareness of the complications associated with neck manipulations in the Southern region of Saudi Arabia.

Data analysis

The data were verified and transmitted to IBM SPSS Statistics for Windows, version 22.0 (released 2013, IBM Corp., Armonk, NY), for analysis after being automatically input into an Excel sheet (Microsoft Corporation, USA). The reliability and clarity of the queries were evaluated in a pilot study conducted on eight randomly selected middle-aged participants. Descriptive and inferential statistical analyses were implemented. Frequencies and percentages of sociodemographic characteristics and other categorical variables were computed and presented in descriptive statistics. The results were also presented in graphical form when necessary to facilitate a more straightforward interpretation. The chi-square test was employed to ascertain the associations between various categorical variables. A p-value of 0.05 or less was considered significant, indicating a 95% confidence interval. IBM SPSS version 27.0.1 was employed to conduct all statistical analyses.

Ethical considerations 

The questionnaire obtained participant consent. King Khalid University Ethics Committee provided ethical approval (approval number 2024-1401). Study activities commenced only after the necessary approval.

## Results

Table 1 shows that a total of 387 respondents took part in the study. The results show a predominance of females, with 278 (71.8%) compared to 109 males (28.2%). Most participants were between 18 and 29 years old (57.4%). In terms of marital status, more than half were single (206, 53.2%). The employment status revealed that many were unemployed (240, 62%), whereas 147 (38%) were employed. Regarding educational level, a large majority had a university education (71.6%), followed by those with secondary education (26.1%).

**Table 1:** Sociodemographic information of the respondents. Sociodemographic information is presented in numbers (n) and percentages (%).

Sociodemographic variables	Category	Frequency(n)	Percentage (%)
Gender	Male	109	28.2
	Female	278	71.8
Age	18-29	222	57.4
	30-39	78	20.2
	40-50	75	19.4
	Above 50	12	3.1
Marital status	Divorced	10	2.6
	Married	167	43.2
	Single	206	53.2
	Widow	4	1.0
Employment status	Not employed	240	62
	Employed	147	38
Educational level	Primary	5	1.3
	Secondary	101	26.1
	University	277	71.6
	Middle	4	1.0

Table 2 indicates that more than a third of the 154 individuals (39.8%) reported experiencing neck pain. The use of blood thinners or aspirin was relatively rare, with only 21 respondents (5.4%) reporting usage. Among the participants, only six individuals (1.6%) had experienced a stroke. Most respondents, 324 (83.7%), had never undergone neck examinations such as TV scans or magnetic resonance imaging, whereas 63 (16.3%) had. In addition, 61 participants (15.8%) reported having been hit in the neck, while 326 (84.2%) had not experienced neck trauma.

**Table 2:** Clinical characteristics of the participants. The data are presented as N, %

Variables	Category	Frequency (n)	Percentage (%)
Do you have persistent neck pain?	No	233	60.2
	Yes	154	39.8
Do you take blood thinners/aspirin?	No	366	94.6
	Yes	21	5.4
Have you ever had a stroke ?	No	381	98.4
	Yes	6	1.6
Have you had neck examinations previously? (TV scan/magnetic resonance imaging)	No	324	83.7
	Yes	63	16.3
Have you been hit in the neck?	No	326	84.2
	Yes	61	15.8

**Figure 1:**
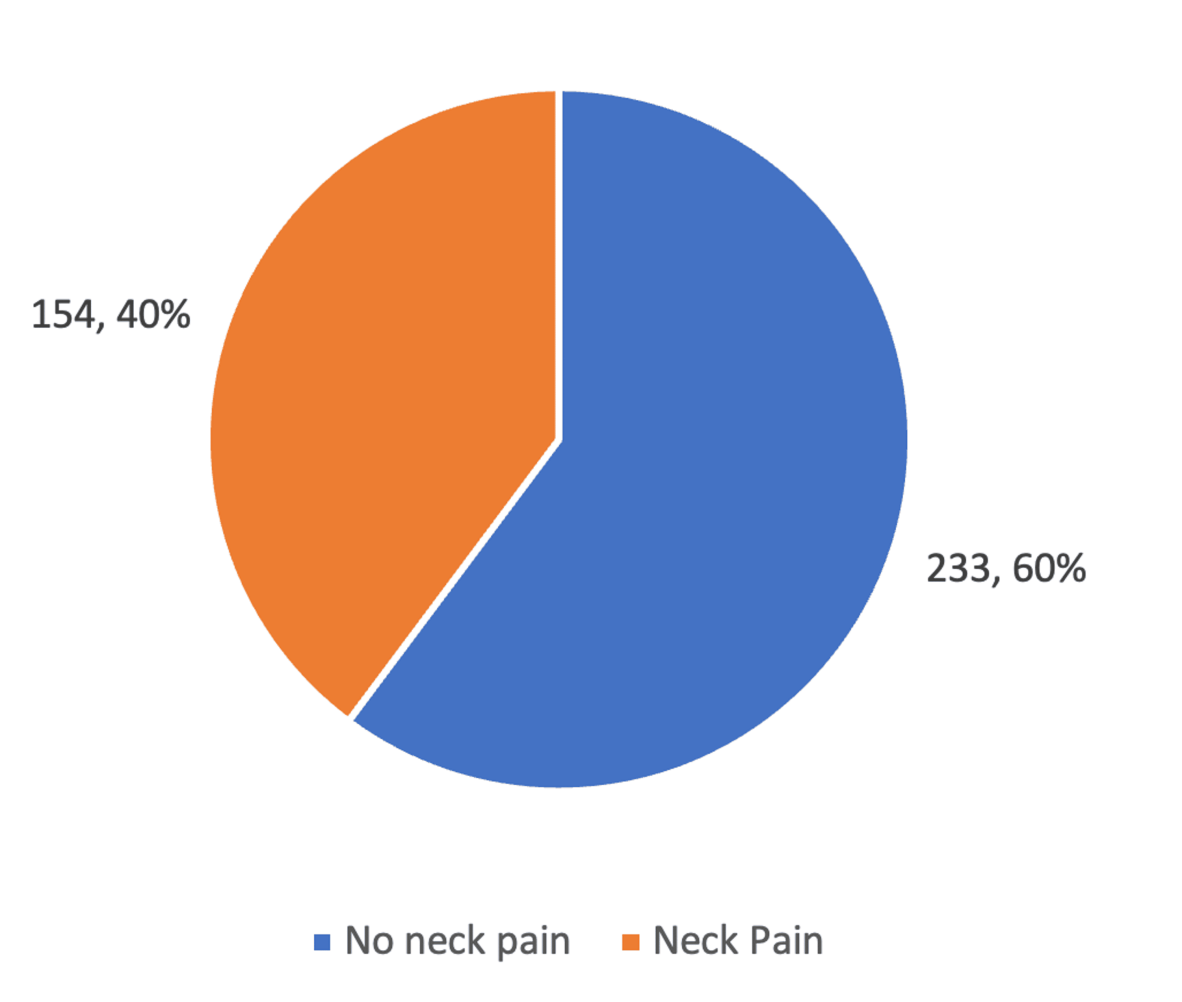
Pie chart showing the prevalence of neck pain among the participants.

Figure 1 shows that the prevalence of neck pain is 39.80% among the participants.

The data in Table 3 reveal that neck massage or cracking is a common practice among respondents, with more than half of 196 individuals (50.6%). The frequency varies among those who perform neck massages, with 103 (52.6%) doing it scarcely, 37 (18.9%). Most respondents (51.0%) asserted that the primary reason for having a massage is to feel comfortable, followed by the need to relieve neck pain (17.9%). Most respondents perform the massage themselves (77, 39.3%), with others receiving it from family or friends (56, 28.6%) or physiotherapists (58, 29.6%). Despite the prevalence of this practice, a significant majority (344, 88.9%) have never visited a chiropractor or masseur, and 86 (22.2%) have had their neck massaged by a barber. Furthermore, 143 respondents (37%) have used a home neck massager. Notably, while 77 individuals (40.8%) claim to know the correct neck massage method, most of this knowledge comes from social media (58, 75.3%).

**Table 3:** Participants practices regarding neck massages. Data are presented as N, %.

Variables	Category	Frequency (n)	Percentage (%)
Have you ever given a neck massage/crack?	No	191	49.4
	Yes	196	50.6
Are you used to doing this? (N=196)	No	100	51.0
	Yes	96	49.0
If yes, how often do you do this? (N=196)	Scarcely	103	52.6
	Daily	27	13.8
	Weekly	37	18.9
	Monthly	26	13.3
Why do you massage/crack the neck? (N=196)	To feel comfortable	100	51.0
	To relieve neck pain	35	17.9
	There is no reason	26	13.3
Who massages/claps for you? (N=196)	By yourself	77	39.3
	From family/friends	56	28.6
	Physiotherapist	58	29.6
	Folk healer	5	2.6
Have you ever visited a chiropractor/masseur?	No	344	88.9
	Yes	43	11.1
Has the barber ever massaged your neck?	No	301	77.8
	Yes	86	22.2
Have you ever used a home neck massager?	No	244	63
	Yes	143	37
Have you ever given someone else a neck massage	No	191	49.4
	Yes	196	50.6
Do you know the correct way to do this? (N=196)	No	116	59.2
	Yes	77	40.8
Where did you learn the correct method? (N = 77)	Social media	58	75.3
	Doctor	5	6.5
	Relatives	1	1.3
	Massager	12	15.6
	Myself	1	1.3

Table 4 shows the participants' knowledge of neck massage complications and post-massage symptoms, revealing that a majority, 271 individuals (70.0%), are unaware of the potential complications, while 116 (30.0%) are informed about them. Among those aware, the most commonly known complications are neck fractures (52, 44.8%), paralysis (47, 40.5%), and stroke (23, 19.8%). Despite this knowledge, 41 individuals (35.3%) would still consider undergoing a neck massage.

**Table 4:** Participants’ knowledge on the complications of neck massage and symptoms after neck massage. Data are presented as N, % (%).

Research question	Category	Frequency (n)	Percentage (%)
Do you know what complications may occur after a neck massage?	No	271	70.0
	Yes	116	30.0
If yes, ‎what complications do you know? (N=116)	Paralysis	47	40.5
	Neck fractures	52	44.82
	Stroke	23	19.8
If you knew about the complications of neck massage, would you still do it? (N=116)	No	63	54.3
	Yes	41	35.3
	Not decided	12	10.3
Did you feel any symptoms after the neck massage?	I did not feel	313	80.9
	Dizziness	20	27.4
	Fatigue	10	13.7
	Headache	24	32.9
	Weakness	13	17.8
If yes, did you need to visit the hospital? (N=73)	No	54	74.0
	Yes	19	26.0

**Figure 2:**
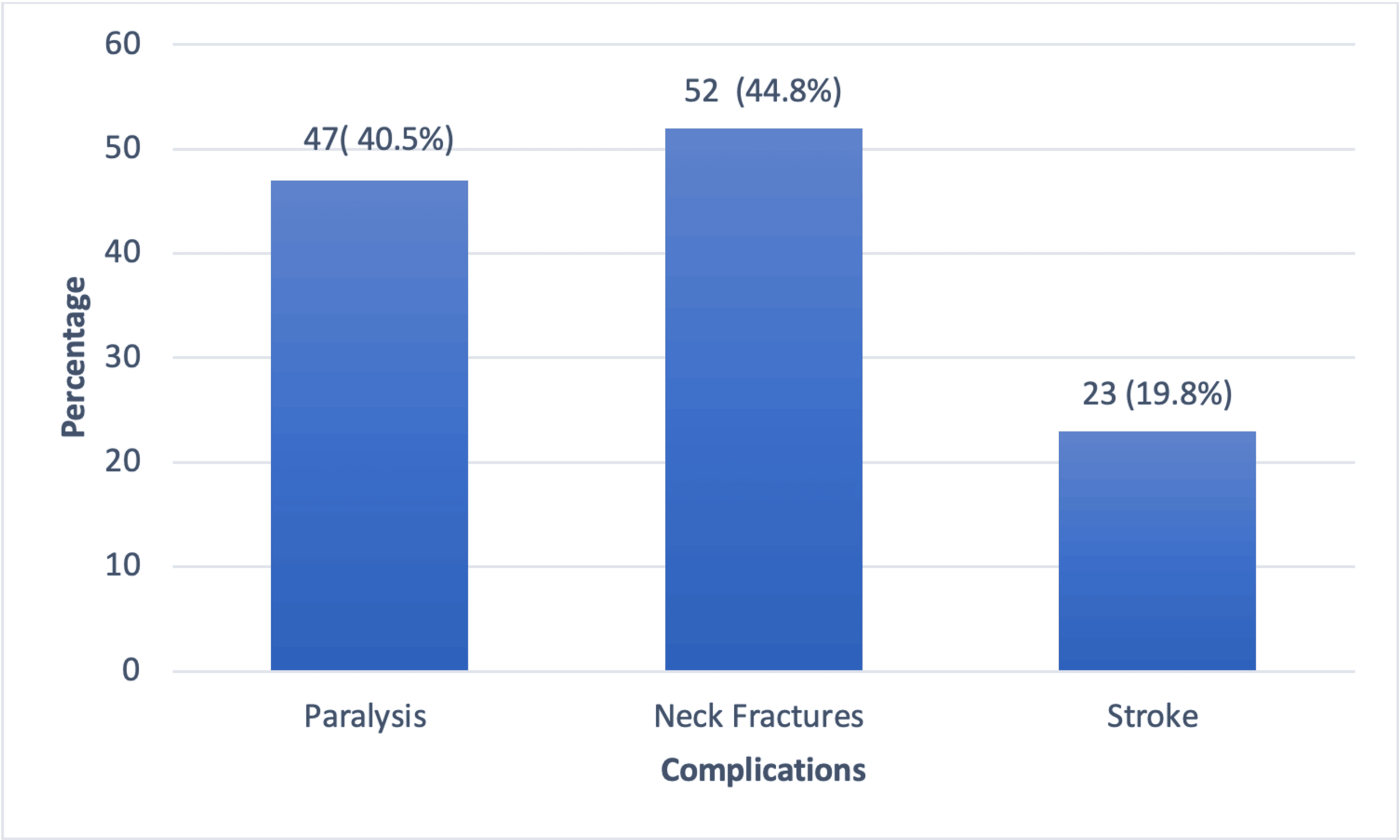
Bar graph depicting the awareness of particular symptoms after a neck massage.

Figure 2 illustrates the complications most commonly recognized by the respondents. Neck fractures were identified by 44.8% of the participants as a potential risk, followed by paralysis, recognized by 40.5%. Stroke was the least frequently acknowledged complication, with 19.8% of the respondents being aware of it.

Table 5 shows that a statistically significant relationship was observed between age and neck pain (p = 0.001). Participants aged 30-39 reported the highest prevalence of neck pain, with 60.3% experiencing this issue, compared to only 32.9% among those aged 18-29 and 40.0% among those aged 40-50. Individuals hit in the neck are more likely to experience neck pain (p < 0.001). Similarly, there was a statistically significant difference between having massage and having neck pain (p < 0.001), with those accustomed to doing it frequently (daily or weekly) showing a significant correlation with neck pain (p < 0.001). Furthermore, those who perform neck massages tend to experience more neck pain (p < 0.001). By contrast, neck pain is not significantly associated with having had a neck massage from a barber (p = 0.345). In addition, gender was not significantly associated with neck pain (p > 0.05), indicating that the likelihood of experiencing neck pain is similar for males and females.

**Table 5:** Association of neck pain with various neck massage practices. A chi-square test was used to find statistical significance. *p < 0.05, considered statistically significant.

Variables	Category	Have neck pain				Chi-square values	P-value
		No		Yes			
		n	%	n	%		
Age	18-29	149	67.1	73	32.9	18.268	0.001
	30-39	31	39.7	47	60.3		
	40-50	45	60.0	30	40.0		
	>50	8	66.7	4	33.3		
Gender	Male	66	60.6	43	39.4	0.007	0.931
	Female	167	60.1	111	39.9		
Have you ever been given a neck massage/crack?	No	145	75.9	46	24.1	38.847	0.001
	Yes	88	44.9	108	55.1		
Are you used to doing this?	No	55	55	45	45	50.214	0.001
	Yes	31	33	63	67		
If yes, how often do you do this?	Daily	8	29.6	19	70.4	58.214	0.001
	Monthly	7	26.9	19	73.1		
	Scarcely	59	57.3	44	42.7		
	Weekly	11	29.7	26	70.3		
Who massages/claps for you?	By yourself	32	44.4	40	55.6	56.031	0.001
	From family/friends	13	50	13	50		
	I do not	148	76.3	46	23.7		
	Physiotherapist	16	57.1	12	42.9		
	Folk healer	1	20	4	80		
Has the barber ever massaged your neck?	No	185	61.5	116	38.5	0.891	0.345
	Yes	48	55.8	38	44.2		
Have you ever used a home neck massager	No	167	68.4	77	31.6	18.695	0.001
	Yes	66	46.2	77	53.8		
Do you know the correct way to do this?	No	71	62.3	43	37.7	10.612	0.001
	Yes	34	44.2	43	55.8		

## Discussion

This particular study aimed to assess the general public’s knowledge and awareness of the complications associated with neck manipulations in the Southern region of Saudi Arabia. The results from this study delineate that the prevalence of neck pain among residents of southern Saudi was 39.8%; this is in support of other literature from other studies, which has found the prevalence of neck pain in general populations to vary widely but is often reported between 20% and 70% [9,10]. In addition, the study found that most participants (83.7%) had never undergone neck examinations, suggesting a potential gap in preventive healthcare measures related to neck manipulations among residents of this region. Furthermore, the study revealed that many participants sought neck massage to relieve pain, with 18.1% citing this as their primary reason for seeking treatment. These results align with findings from Beliveau PJ et al., which identified neck pain as the second most common reason people seek neck massage [9].

Interestingly, the study revealed that most participants (88.9%) had never visited a chiropractor or masseur. This suggests that many individuals in this study relied on informal or self-taught techniques for performing neck massage. This reliance on unprofessional methods can complicate the follow-up of patient histories, especially when serious symptoms develop after neck massage [11]. These findings are supported by Bogduk, who noted in his study that no data determines the percentage of pain-free patients following manual therapy for neck pain [11].

In terms of knowledge regarding the correct way to execute neck massage, the results from the study designate that half of the respondents in this study (50.6%) had given a neck massage, but only (19.9%) knew the appropriate way to undertake it, with a majority attesting that they learned the technique from social media (75.3%). The low awareness of correct methods is consistent with a similar study by Cook et al., which pinpointed minimal formal training, if any, and reliance on informal sources like social media for health practices [12]. The higher percentage (75.3%) of the population embracing social media as a source for learning neck massage techniques aligns with broader trends of increasing reliance on digital platforms for health information [13,14].

Regarding risks, most (70%) were unaware of potential complications. Among those who were aware, neck fractures (44.8%) and paralysis (40.5%) were the most commonly identified complications. This lack of awareness is consistent with research on chiropractic manipulations by Hurwitz et al., which highlighted poor public awareness of the risks and potential adverse effects of such treatments and recommended targeted training to improve awareness about the risks and potential complications associated with neck manipulations [15].

The study found a statistically significant association between neck pain and various neck massage practices. Specifically, there was a significant relationship between neck trauma and neck pain (p = 0.001), which contrasts with a similar study by Almalki et al. conducted in Saudi Arabia that did not show any statistically significant differences [3]. The differences could be attributed to different sample populations or regional practices. Interestingly, age also plays a significant role in influencing neck pain prevalence. This relationship suggests that as individuals age, the susceptibility to or reporting neck pain may increase, potentially due to age-related degeneration or variations in physical activity levels and health awareness. In addition, other neck massage practices, including receiving neck massages and the frequency of these massages, were significantly associated with neck pain. The study also identified a significant association between neck pain and knowledge of correct massage techniques (p = 0.005).

This study encountered several inherent limitations. Being a cross-sectional survey, it could not determine causal relationships between variables. In addition, the use of online questionnaires relied on self-reported responses, which lack direct verification and may introduce biases. The generalizability of the findings is also limited, as the study was restricted to the southern region. Moreover, while this study focused on the public's awareness of severe complications such as paralysis, fractures, and stroke associated with neck manipulations, certain symptoms like aggravation of pain, sensory loss, and bladder dysfunction were not explicitly addressed. This limitation stems from the questionnaire design, which prioritized severe outcomes commonly reported in the literature. Future studies should consider expanding the scope to include these symptoms, as they may provide a more comprehensive understanding of the spectrum of complications related to neck manipulations.

## Conclusions

This study aimed to evaluate public knowledge and awareness of complications associated with neck manipulations in the southern region of Saudi Arabia. The findings reveal a significant lack of awareness among the general population regarding potential risks despite the prevalence of neck manipulation practices. The study concluded that most participants needed better general knowledge about possible complications, especially concerning severe outcomes such as stroke. The most common reason for seeking neck manipulations was comfort, followed by pain relief. Surprisingly, most university-educated participants also demonstrated poor knowledge about potential risks. Most participants were positive toward neck manipulations, with many practicing it regularly. However, this positive perception did not match a comprehensive understanding of associated risks. The findings from this study will contribute to the existing literature. They can be used to plan strategies to educate the public about neck manipulations' benefits, risks, and complications. At the same time, it also serves as a valuable source of information to support further studies.

## Appendices

Study questionnaire

**Table 6:** Sociodemographic Information.

Sociodemographic variables	Category
Gender	Male
	Female
Age	18-29
	30-39
	40-50
	Above 50
Marital status	Divorced
	Married
	Single
	Window
Current living country	Saudi Arabia
	Other:
Living region	Eastern region
	Central region
	Western region
	Northern region
	Southern region
Marital status	Divorced
	Married
	Single
	Window
Employment status	Not employed
	Employed
Educational level	No formal education
	Elementary school
	Middle school
	High school
	Technical education
	Bachelor’s degree
	Master’s degree
	Doctoral degree

**Table 7:** Awareness and experience with neck manipulations.

Variables	Category
Do you have neck pain?	No
	Yes
Do you take blood thinners/aspirin?	No
	Yes
Have you ever had a stroke?	No
	Yes
Have you had neck examinations previously? (US/MRI)	No
	Yes
Have you been hit in the neck?	No
	Yes
Have you ever given a neck massage/crack?	No
	Yes
Are you used to doing this?	No
	Yes
If yes, how often do you do this?	Scarcely
	Daily
	Weekly
	Monthly
Why do you massage/crack the neck?	To feel comfortable
	To relieve neck pain
	There is no reason
Who massages/claps for you?	By yourself
	From family/friends
	Physiotherapist
	Folk healer
Have you ever visited a chiropractor/masseur?	No
	Yes
Has the barber ever massaged your neck?	No
	Yes
Have you ever used a home neck massager?	No
	Yes
Have you ever given someone else a neck massage	No
	Yes
Do you know the correct way to do this?	No
	Yes
Where did you learn the correct method?	Social media
	Doctor
	Relatives
	Massager
	Myself

**Table 8:** Complications and symptoms.

Question	Category
Do you know what complications may occur after a neck massage?	No
	Yes
If yes, ‎what complications do you know?	Paralysis
	Neck fractures
	Stroke
If you knew about the complications of neck massage, would you still do it?	No
	Yes
	Not decided
Did you feel any symptoms after the neck massage?	I did not feel
	Dizziness
	Fatigue
	Headache
	Weakness
If yes, did you need to visit the hospital?	No
	Yes

## References

[1] Borghouts J, Koes B, Vondeling H, Bouter L (1999). Cost-of-illness of neck pain in the Netherlands in 1996. Pain.

[2] Hurwitz EL, Chiang LM (2006). A comparative analysis of chiropractic and general practitioner patients in North America: findings from the joint Canada/United States Survey of Health, 2002-03. BMC Health Serv Res.

[3] Almalki S, Almaghnem MS, Alkulaib SS, Alhafith RF (2024). General public awareness and knowledge of neck maneuvers complications in Saudi Arabia: a cross-sectional study. Int J Med Dev Ctries.

[4] Yeomans S (2024). Spine-health | Chiropractic manipulation for the cervical spine. Chiropractic Manipulation for the Cervical Spine. Spine-health.

[5] Stevinson C, Honan W, Cooke B, Ernst E (2001). Neurological complications of cervical spine manipulation. J R Soc Med.

[6] Britt TB, Agarwal S (2024). Vertebral artery dissection. StatPearls [Internet].

[7] Norris JW, Beletsky V, Nadareishvili ZG (2000). Sudden neck movement and cervical artery dissection. CMAJ.

[8] Senstad O, Leboeuf-Yde C, Borchgrevink C (1996). Predictors of side effects to spinal manipulative therapy. J Manipulative Physiol Ther.

[9] Beliveau PJ, Wong JJ, Sutton DA, Simon NB, Bussières AE, Mior SA, French SD (2017). The chiropractic profession: a scoping review of utilization rates, reasons for seeking care, patient profiles, and care provided. Chiropr Man Therap.

[10] Kazeminasab S, Nejadghaderi SA, Amiri P (2022). Neck pain: global epidemiology, trends and risk factors. BMC Musculoskelet Disord.

[11] Bogduk N (2003). Spinal manipulation for neck pain does not work. J Pain.

[12] Cook AJ, Wellman RD, Cherkin DC, Kahn JR, Sherman KJ (2015). Randomized clinical trial assessing whether additional massage treatments for chronic neck pain improve 12- and 26-week outcomes. Spine J.

[13] Kettering V (2016). Social healing: increasing awareness of complementary and alternative medicine treatment options through empowerment technology. https://www.proquest.com/openview/724cc256c0a4b4517335358451f8e8ee/1?pq-origsite=gscholar&cbl=18750.

[14] Huo J, Desai R, Hong YR, Turner K, Mainous AG 3rd, Bian J (2019). Use of social media in health communication: findings from the Health Information National Trends Survey 2013, 2014, and 2017. Cancer Control.

[15] Hurwitz EL, Morgenstern H, Vassilaki M, Chiang LM (2005). Frequency and clinical predictors of adverse reactions to chiropractic care in the UCLA neck pain study. Spine (Phila Pa 1976).

